# Synthetic short peptide mimicking IsdB and IsdH conserved motifs selectively bind and enable detection of *Staphylococcus aureus* infection

**DOI:** 10.3389/fimmu.2026.1813440

**Published:** 2026-05-20

**Authors:** Taiwo Samuel Agidigbi, Irvin Oh

**Affiliations:** Department of Orthopedics and Rehabilitation, Yale University, New Haven, CT, United States

**Keywords:** humoral immunity, ISDB, IsdH, musculoskeletal infection, *Staphylococcus aureus*

## Abstract

**Background:**

*Staphylococcus aureus* (*S. aureus*) is the most common pathogen in musculoskeletal infections (MSKI). Application of antibody responses to *S. aureus* virulence factors have suggested to improve diagnostic specificity. Iron acquisition plays a crucial role in *S. aureus* pathogenesis. The bacterium scavenges iron from the host by expressing cell surface receptors that bind heme and hemoglobin through the iron-regulated surface determinant (Isd) system. These heme and hemoglobin binding motifs are essential for bacterial survival and virulence. In particular, the highly conserved region of the near iron transport (NEAT) domain may serve as a target for diagnostic applications.

**Methods:**

Based on our review of multiple IsdB and IsdH amino acids sequences, we identified a highly conserved near iron transport (NEAT) domains that are unique to *S. aureus*. We designed and synthesized two short peptides: IsdB-NEAT2, containing the heme binding motifs, and IsdH-NEAT2, containing the hemoglobin binding motif. Each represents the most highly conserved region of the IsdB and IsdH. We utilized infected tissue culture and *Staphylococcal* complement inhibitor (SCIN) as an adjunct reference. We used enzyme-linked immunosorbent assay (ELISA) with three antigens; IsdB-NEAT2, IsdH-NEAT2, and SCIN to measure reactivity of sera from three groups: patients with *S. aureus* MSKI (N = 20), patients with MSKI due to other microbial etiologies (N = 20), and healthy controls (N = 21). Circulating immunoglobulins IgM and IgG were measured. Cytokines production by peripheral blood mononuclear cells (PBMC) following stimulation with IsdB-NEAT 2 and IsdH-NEAT 2 antigens were comparatively analyzed against PBMCs from a control group not challenged with *S. aureus*-specific antigens.

**Results:**

Sera from *S. aureus*-infected patients showed significantly elevated ELISA optical density (OD) reactivity with IsdB-N2, IsdH-N2, and SCIN (p <0.0001; AUC at 95% confidence interval [CI] > 0.99) respectively compared to healthy controls. Comparisons with other MSKI, sera from *S. aureus*-associated MSKI yielded significant differences for all three antigens IsdB-N2, IsdH-N2, and SCIN (p <0.0001; AUC at 95% CI were = 0.995, = 0.885 and = 0.920) respectively. Total serum IgM and IgG were markedly higher in *S. aureus* patients vs. healthy controls (both p = 0.0001). Antigen-specific IgM and IgG levels were significantly elevated in the *S. aureus* group vs. healthy controls for all three antigens (IgM: IsdB-N2, p = 0.0057; IsdH-N2, p = 0.0040; SCIN, p = 0.0011; IgG: IsdB-N2, p = 0.0499; IsdH-N2, p = 0.0207; SCIN, p = 0.0068). Both peptides stimulated significant cytokine production by PBMCs compared with the untreated PBMCs, inducing *IL-6, IL-8, IL-10, IL-1β, MCP-1, TNF-α, EGF*, and *RANTES*.

**Conclusions:**

Our findings indicate that humoral responses to the novel *S. aureus* antigens (IsdB-NEAT2, IsdH-NEAT2) were able to differentiate *S. aureus* associated MSKI from other infections, and from healthy controls. Both IsdB-NEAT2 and IsdH-NEAT2 stimulated pro-inflammatory and chemotactic cytokines, confirming that these short-sequenced antigenic motifs activated the innate immune system. This study presents a novel, shortened, and highly conserved component of synthetic Isd peptides, that mimics the functional heme and hemoglobin binding motifs of *S. aureus* Isd proteins, which could be utilized for diagnosis of *S. aureus* MSKI.

## Introduction

*Staphylococcus aureus* (*S. aureus*) remains a leading cause of community and healthcare-associated infections worldwide, responsible for a spectrum of diseases ranging from skin and soft tissue infections in orthopedics to life-threatening bacteremia and endocarditis (1–6). Rapid, specific and cost-effective identification of *S. aureus* from clinical specimens is critical to institute timely therapeutic interventions and infection control strategies. Conventional laboratory methods (culture, biochemical tests) are often time-consuming, and not always reliable (7–9); molecular assays such as PCR and rapid immunochromatographic and lateral-flow tests offer faster turnaround but can be limited by cost, infrastructure requirements, sensitivity in complex matrices, and the need for high-quality reagents or antibodies. These limitations motivate the need for the development of new diagnostic probes that combine rapidity, accuracy, and manufacturability (10–13).

A promising molecular target for *S. aureus* detection is its iron-acquisition machinery (14, 15). Iron is essential for bacterial growth but is tightly sequestered by the host (nutritional immunity), forcing pathogens to evolve specialized uptake systems (15). *S. aureus* primarily acquires iron via two complementary strategies: production of siderophores (staphyloferrins) and direct capture of heme from host hemoproteins (14–17). The Iron-regulated surface determinant (Isd) system mediates heme capture and relay across the cell wall into the cytoplasm and is upregulated under iron limitation encountered during infection (18, 19). Because Isd proteins are surface exposed and functionally conserved across clinical isolates, they have been explored both as virulence factors and as potential diagnostic and vaccine targets (18–20).

The Isd system comprises nine proteins whose expression is collectively induced under iron-limited conditions (21–23). The principal function of the Isd machinery is to extract hemoglobin (Hb) and mediate its translocation into the bacterial cell (15, 22, 23). Among these components, IsdA, IsdB, IsdC, and IsdH are covalently anchored to the cell wall peptidoglycan, thereby displaying their functional domains on the cell surface (24, 25). Each protein contains one or more structurally conserved near-iron transporter (NEAT) domains that facilitate the binding of Hb and heme. Within the Isd family, IsdB and IsdH have attracted particular attention (4–6). Structural and functional studies indicate that IsdB acts as a hemoglobin receptor and participates directly in heme extraction, whereas IsdH (also called HarA/IsdHE) contributes to hemoglobin binding and heme transfer along the relay (19, 23–25). The extracellular localization and essential role of these receptors in heme scavenging make them attractive antigenic targets for immunoassays and surface-directed probes; however, full-length Isd proteins are large, complex and can be heterogeneous between strains, complicating recombinant expression and increasing production cost for diagnostics based on whole proteins or long polypeptides (26, 27).

Short peptides present several practical advantages such as diagnostic reagents (28, 29). Carefully chosen peptide epitopes can reproduce the immunodominant or receptor-binding motifs of larger proteins while being much simpler and cheaper to synthesize, chemically define, and store (28, 29). Peptide-based probes have been used successfully for serological assays, epitope mapping, and development of peptide microarrays and lateral-flow formats; they often reduce batch variability and enable high-throughput manufacture (28, 29). Importantly, when a conserved functional motif is known, a short peptide that mimics that motif can preserve specificity while permitting facile chemical synthesis and engineering (e.g., conjugation, multimerization, or immobilization) for incorporation into point-of-care platforms (28–32).

Building on these considerations (28–32), we synthesized short peptides that contain conserved motifs predicted to capture the key molecular signature of *S. aureus* Isd-mediated hemoglobin/heme interaction. Here we present the biochemical rationale for selecting the conserved motifs, the peptide design and synthesis, and initial evaluation of its potential to discriminate *S. aureus* from healthy control and related bacteria, thereby providing a foundation for a rapid, affordable diagnostic alternative to conventional approaches (33, 34).

Objective: 1. To develop and evaluate shortened synthetic peptides derived from key functional conserved motifs of the *S. aureus* iron-regulated surface determinant (Isd) proteins in the serum of patients with *S. aureus* musculoskeletal infection (MSKI) for potential diagnostic application. **2**. to evaluate whether synthesized Staphylococcus aureus–derived peptides (IsdB-NEAT2, IsdH-NEAT2, SCIN) exhibit selective and high-avidity binding to antibodies present in sera from individuals with confirmed *S. aureus* infection, compared with healthy controls and patients with musculoskeletal infections (MSKI) of alternative etiology. 3. To assess the immunogenicity of the peptide by evaluating peripheral mononuclear blood cells (PBMCs) activation, which reflects potential host immune responses.

## Methods

### Patients’ enrollment

During this study, we enrolled twenty (n=20) patients (age between 36–78 years) who presented with symptoms and signs of chronic and acute MSKI (periprosthetic joint infection, septic arthritis of the native joint or foot and ankle infection) whose intra-operative cultures identified *S. aureus* monomicrobial infection that required urgent clinical attention. We also recruited twenty (n=20) patients with other non-*S. aureus* associated with MSKIs polymicrobials (8) or monomicrobials (12), the etiologic agents included *Psuedomonas aeruginosa, Staphylococcus lugdunensis, Enterococcus faecalis, group G streptococcus, group A Streptococcus Bacteroides fragilis* and *Corynebacterium striatum.* Twenty-one (n=21) non-infected individuals as healthy control (these are people presented with either accident, tendon rupture, foot equinovarus deformity, peroneus brevis tear, fracture or non-infection related symptoms and disclosed no prior use of antibiotics within 3 months or underline ailments) (**Table 1**). Patients with a confirmed history of antibiotic use, comorbidities, or inflammatory-related conditions were excluded from the study. Blood samples were drawn to obtain serum, while PBMC were obtained by gradient density using Ficoll-Paque.

**Table 1:** Demographics and clinical characteristics of study subjects.

Group	N	Age (mean)	Sex (M/F)	Infection site/MSKI type
*S. aureus* MSKI	20	58 ± 20	13/7	Ankle, knee, and foot Osteomyelitis, native joint septic arthritis, prosthesis joint infections.
Other MSKI	20	60 ± 21	12/8	Ankle, knee, foot, and hip. *Streptococcus mitis*, Group B *Streptococcus* (GBS), Group A *Streptococcus*, *Enterococcus faecalis*, *Bacteroides fragilis*, *Staphylococcus lugdunensis*, and *Psuedomonas aeruginosa*
Healthy controls	21	57 ± 13	9/12	Foot and Ankle deformities and injuries, including tendon ruptures and fractures.

### Peptide sequences selection and synthesis

Through analysis of multiple IsdB and IsdH amino acids sequences (4, 25, 26, 35–38), we did multiple sequences comparative study, we identified a common epitope repeatedly occurring in most sequences of Isd with highly conserved motifs corresponding to NEAT-2 domains, which are unique to *S. aureus*. Guided by these findings, we designed and synthesized two short peptides, IsdB-NEAT2 heme binding domains (KTIDYGQYHVRIVDKEAFTKANTDKS) (**Supplementary Figure 1**) and IsdH-NEAT2 hemoglobin biding domains (VYEGDKKLPVELVSYDSD-KDYAYIRFPVSNGTREVKIVSS) peptides (**Supplementary Figure 2**) in collaboration with GenSript. Commercially available SCIN antigen was purchased from Biorbyt manufacturer (Lot 04297) as an adjunct reference.

### Serological test/avidity assay

(1) Antigen-antibody avidity was evaluated by enzyme-linked immunosorbent assay (ELISA). Serum samples were diluted 1:500 in phosphate-buffered saline (PBS) and incubated overnight at 4 °C in 96-well plates to allow adsorption/coating. Plates were then blocked with PBS containing 1% bovine serum albumin (BSA) for 2 h at room temperature. Following blocking, biotinylated labeled (EZ-Link™ Sulfo-NHS-Biotin, ThermoFisher Scientific, lot YJ377263) recombinant peptides (IsdB-NEAT2, IsdH-NEAT2, and SCIN; each at 5 μg/mL) were added and incubated for 2 h at room temperature. Plates were washed thoroughly between each step with PBS containing 0.05% Tween-20. Bound antigen–antibody complexes were detected using horseradish peroxidase (HRP)-conjugated streptavidin, followed by the addition of substrate solution. The reaction was stopped with acid stop solution, and optical density (OD) was measured at 450 nm using a microplate reader. (2) serum samples were serially diluted in dilution buffer (without peptides) and added to 96 well plates IgM (human IgM, Abcam, AB137982, lot 1075029-4) and IgG (human IgG, Abcam, AB195215, lot 2101056573), total circulating IgM and IgG were evaluated according to manufacturer instructions. (3) Serum samples (control, *S. aureus* infection and other MSKI etiology) were diluted 1:1000 in phosphate-buffered saline (PBS), Biotin-labeled recombinant peptides (IsdB-NEAT2 and IsdH-NEAT2) were added to the diluted serum at a final concentration of 5 µg/mL and incubated overnight at 4 °C in 1.5 mL microcentrifuge tubes to allow immune complex formation. Following incubation, the serum–peptide mixtures were transferred to enzyme-linked immunosorbent assay (ELISA) plates pre-coated with anti-human IgM or IgG and OD values were measured according to manufacturer’s instructions.

### Cell viability-MTT assay

The MTT (3- (4, 5-dimethylthiazol-2-yl) −2,5-diphenyltetrazolium bromide) assay was performed to assess the cytotoxicity of our test compound. IsdB-NEAT2 and IsdH-NEAT2 were diluted in RPMI 1640 supplemented with BSA at varying concentration, and 16 PBMCs samples, four (4) from each group (from the same donors used for serological analysis) were seeded at a density of 1 × 10^5^ cells/well in a 96 well plate. The PBMCs were incubated with serial dilutions of the test compound in triplicate at 37°C in a 5% CO_2_ atmosphere with 100% humidity for 24 hours, the experiment was performed independently in 3 different times. Twenty μl of a 5 mg/ml MTT solution was added to each well and the plate was further incubated at 37 °C for 4 hours. Thereafter the medium was aspirated and 200 μl of DMSO was added to each well. The plate was placed on a shaker to dissolve the formazan crystals, thereafter the absorbance was determined spectrophotometrically at 570 nm on an ELX800 UV universal microplate reader (Bio-Tek Instruments Inc., Vermont, USA).

### Trypan blue proliferative assay

IsdB-NEAT2 and IsdH-NEAT2 were diluted in RPMI 1640 at 5000 ng/mL, and PBMCs were seeded at a density of 1 × 10^5^ cells/well in a 96 well plate. The PBMCs were incubated with serial dilutions of the test compound in triplicate at 37°C in a 5% CO_2_ atmosphere with 100% humidity. After 72 hours, cells were harvested in culture media, and 10 μL volume of the cell suspension was mixed with an equal volume (10 μL) of trypan blue stain. The mixture was then immediately loaded onto a hemocytometer to visualize and count cells, allowing determination of cell viability.

### Pro-inflammatory cytokine assay

PBMCs from healthy patients were seeded at a density of 2 × 10^6^ cells/well in a 24 well plate in the presence or absence of 5000 ng/mL of IsdB-NEAT2 and IsdH-NEAT2 each diluted in RPMI 1640. PBMCs from patients with *S. aureus* infection were cultured without stimulation. After 24 hours, culture media samples were collected and incubated with a membrane containing capture antibodies for a panel of cytokines (biotinylated human cytokine antibody array, lot 1135186-1; Abcam). Cytokines levels were then visualized using a detection system (ChemiDoc™ Touch Imaging System; BioRad) and quantified with ImageJ software, following the manufacturer’s instructions.

### Result analysis

Overall, OD absorbance was read at 450 nm and 750 nm for the MTT assay. IgG and IgM concentrations were determined by extrapolating from the standard curve, while cytokine activation was quantified by densitometry using ImageJ. Comparisons between groups were initially assessed using the non-parametric Mann–Whitney U test in GraphPad Prism version 10 (GraphPad Software, San Diego, CA, USA) and ROC curve analysis were performed using R to assess the overall accuracy represented by the area under the ROC curve. ROC curve analysis was performed to evaluate the diagnostic performance of serum biomarkers (IsdB, IsdH, and SCIN) in distinguishing *Staphylococcus aureus* infections from controls and infections of other etiologies. All analyses were conducted using R statistical software (version 4.5.2), employing relevant packages such as *pROC* for curve generation and performance estimation. For each biomarker, ROC curves were constructed by plotting sensitivity against 1 − specificity across a range of threshold values. The area under the ROC curve (AUC) was calculated as a measure of overall diagnostic accuracy, with corresponding 95% confidence intervals (CIs) estimated using the nonparametric method.

## Results

### Immunoreactivity of IsdB-NEAT2 and IsdH-NEAT2 Antigens with Sera from *S. aureus* MSKI patients

**Figure 1:**
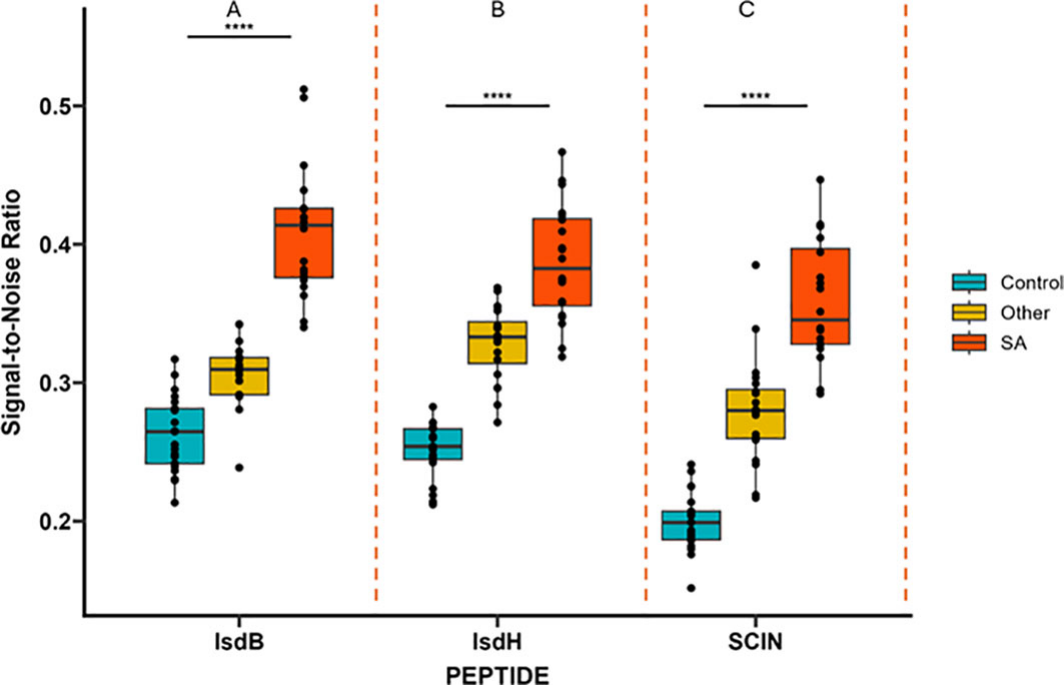
Serum antibody responses to *Staphylococcus aureus* antigens in patients with *S. aureus* MSKI, other MSKI, and healthy controls. Enzyme-linked immunosorbent assays (ELISAs) were performed to measure the Optical density (OD) of antibody reactivity to staphylococcal antigens **(A)** IsdB-NEAT2, **(B)** IsdH-NEAT2 and **(C)** SCIN (****p<0.0001).

To evaluate the ability of our short, synthesized Isd proteins peptides to detect circulating *Staphylococcus aureus* antibodies during infection, sera from patients with *S. aureus* associated with MSKI, individuals with other MSKI, and non-infectious healthy controls were analyzed for immunoreactivity against IsdB-NEAT2, IsdH-NEAT2, and SCIN antigens using ELISA. Sera from patients with confirmed *S. aureus* MSKI exhibited higher antibody levels compared with healthy controls (IsdB-N2, IsdH-N2, and SCIN, p <0.0001) respectively (**Figures 1A–C**). When compared with sera from patients with other non-*S. aureus* MSKI, the *S. aureus* MSKI group showed elevated antibody responses to all three antigens (IsdB-N2, IsdH-N2, and SCIN, p <0.0001) respectively. Together, these data indicate that IsdB-NEAT2 and IsdH-NEAT2 reliably detect circulating anti-*S. aureus* antibodies and effectively discriminate *S. aureus* MSKI from both non-staphylococcal MSKI and healthy controls, supporting their potential as diagnostic serological biomarkers.

### Diagnostic accuracy (OD value) cutoff point of IsdB, IsdH and SCIN

**Figure 2:**
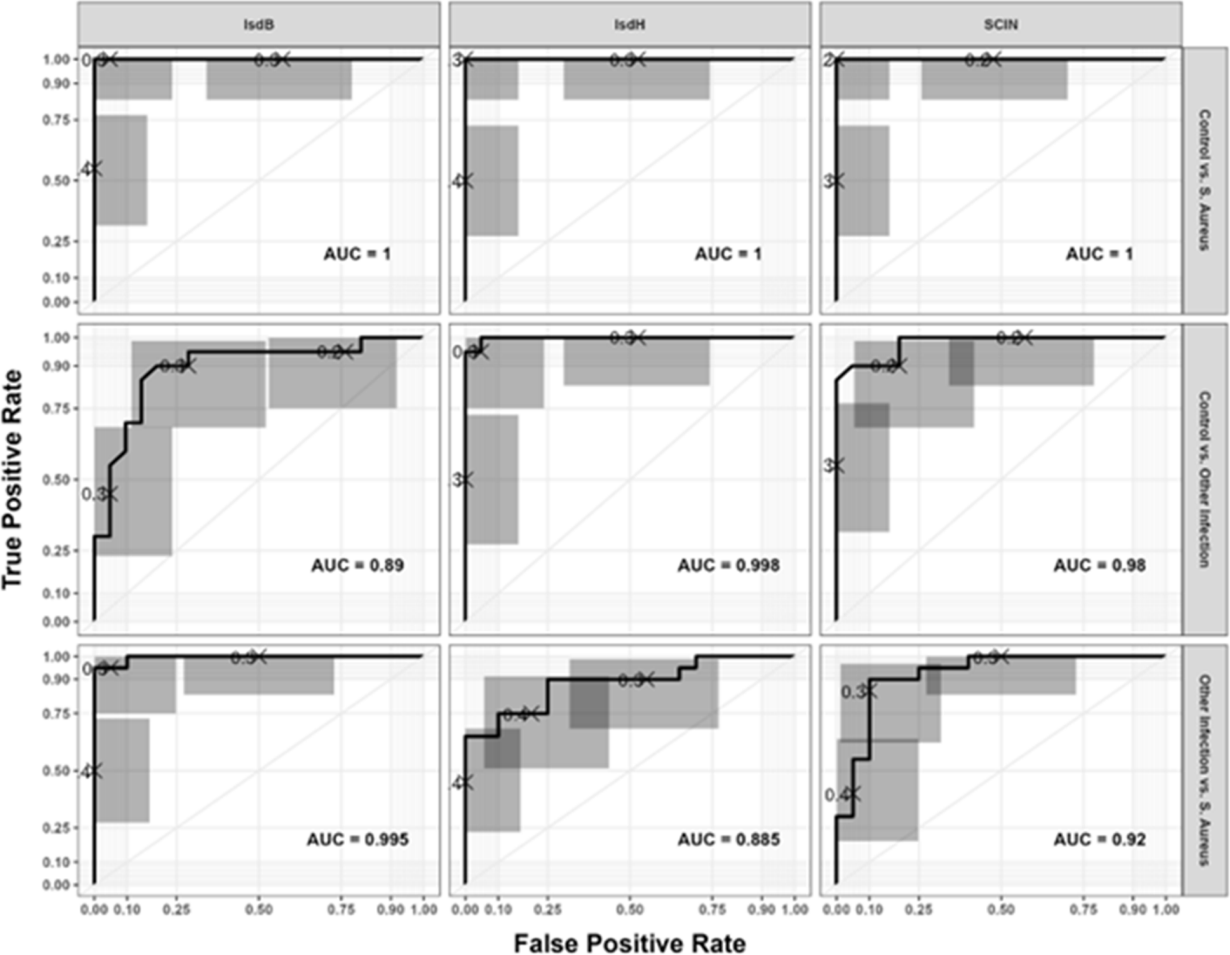
ROC analysis of serum biomarkers for diagnosing *Staphylococcus aureus* infection. ROC curves for IsdB, IsdH, and SCIN show excellent discrimination between *S. aureus* infection and healthy controls (AUC > 0.99, 95% CI). For differentiation from other infections, AUCs were 0.995 (IsdB), 0.885 (IsdH), and 0.920 (SCIN).

Comparative analyses were performed for two classification groups: (1) *S. aureus* infection versus healthy controls, and (2) *S. aureus* infection versus infections of other etiologies. The AUC values for serum IsdB, IsdH, and SCIN demonstrated excellent discriminatory performance in distinguishing *S. aureus* infections from controls (all 95% CI > 0.99). For differentiation between *S. aureus* infections and other etiologies, the AUC values were 0.995 for IsdB, 0.885 for IsdH, and 0.920 for SCIN (**Figure 2**). Optimal cutoff values for each biomarker were determined using the Youden J index (J = sensitivity + specificity − 1), which identifies the threshold that maximizes the combined sensitivity and specificity. Based on this approach, the cutoff values for distinguishing *S. aureus* infection from controls were 0.3400 (IsdB), 0.3187 (IsdH), and 0.2920 (SCIN). For distinguishing *S. aureus* infection from other etiologies, the optimal cutoff values were 0.3440 (IsdB), 0.3580 (IsdH), and 0.3183 (SCIN).

### General humoral immune response to *S. aureus* MSKI

**Figure 3:**
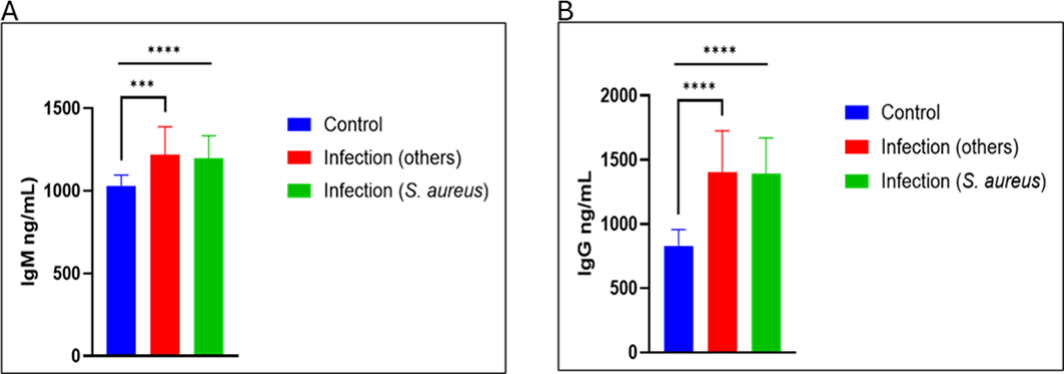
General humoral immune response in patients with *Staphylococcus aureus* musculoskeletal infections. **(A)** Circulating IgM and **(B)** IgG antibody levels were measured in patients with confirmed *S. aureus* MSKI, infection of other etiology and healthy controls. Both IgM and IgG concentrations were significantly elevated in infected individuals (***p < 0.0001 for both comparisons) (***p < 0.001 and ****p < 0.0001).

Circulating IgM and IgG antibody levels were measured in the sera of patients with confirmed *S. aureus*, MSKI of other etiology and compared with those of healthy controls. As shown in **Figure 3**, both IgM and IgG concentrations were significantly elevated in patients with MSKI of other etiologies and *S. aureus* compared to healthy controls. For IgM, levels were increased in cases of other MSKI etiologies (*p* < 0.0005) and *S. aureus* infection (*p* < 0.0001). Similarly, IgG concentrations were significantly higher in both other MSKI etiologies and *S. aureus* infection groups (*p* < 0.0001 for each). Although low baseline levels of IgM and IgG were detectable in healthy controls, antibody responses were markedly elevated in patients with confirmed infections. Notably, the IgG response exceeded the IgM response, consistent with expected humoral immune kinetics in which early IgM production is followed by a more robust and sustained IgG response after immune sensitization. Elevated IgG levels therefore indicate an active, ongoing adaptive immune response to MSKI.

### Detection of *S. aureus* specific antibodies using IsdB-NEAT2 and IsdH-NEAT2 peptides

**Figure 4:**
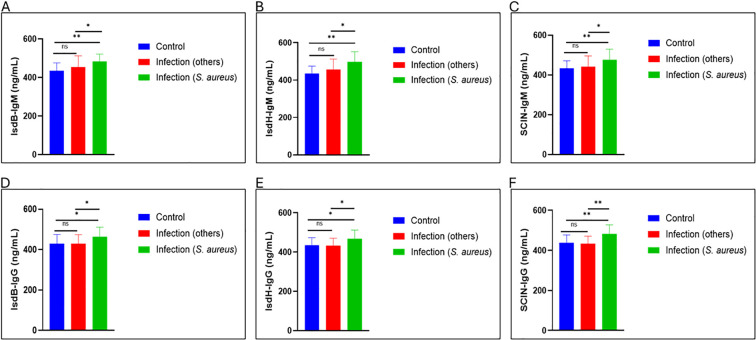
Antigen-specific serological detection of *Staphylococcus aureus*. Sera pre-treated with IsdB-NEAT2, IsdH-NEAT2 peptides and SCIN control showed higher IgM and IgG levels in *S. aureus* MSKI patients than in healthy controls, confirming antigen-specific responses. **(A)** IsdB NEAT2 IgM; **(B)** IsdH NEAT2 IgM; **(C)** SCIN IgM; **(D)** IsdB NEAT2 IgG; **(E)** IsdH NEAT2 IgG; **(F)** SCIN IgG. *p* < 0.05. (*p < 0.05, and **p < 0.01)

Analysis of circulating general immunoglobulins demonstrated that IsdB-NEAT2, IsdH-NEAT2 peptides, and SCIN effectively detected elevated serum IgM and IgG antibodies in individuals with *S. aureus* MSKI. To assess the avidity responses of IsdB and IsdH antibodies in the sera of patients with S. aureus infection compared to others, an ELISA-based avidity test was employed to quantify peptide–antibody interactions, using biotinylated recombinant peptides to probe immobilized serum immunoglobulins. Optical density (OD_450_) serves as a surrogate for binding strength, with higher signals reflecting increased avidity and specificity. Although measurement of total circulating IgM and IgG was performed in parallel to control for inter-sample variability in immunoglobulin levels (**Figure 3**), but to ensure that observed differences are peptide-bound reactivity rather than changes in antibody abundance. Our results confirm that both IsdB-NEAT2 and IsdH-NEAT2 peptides can detect antibodies directed against *S. aureus* compared to MSK of other etiology and health control (**Figure 4**). This finding highlights the potential of these peptides as tools for the serological detection of *S. aureus* MSKI.

### Proliferative/cytotoxicity activity of IsdB-NEAT2 and IsdH-NEAT2 peptides

**Figure 5:**
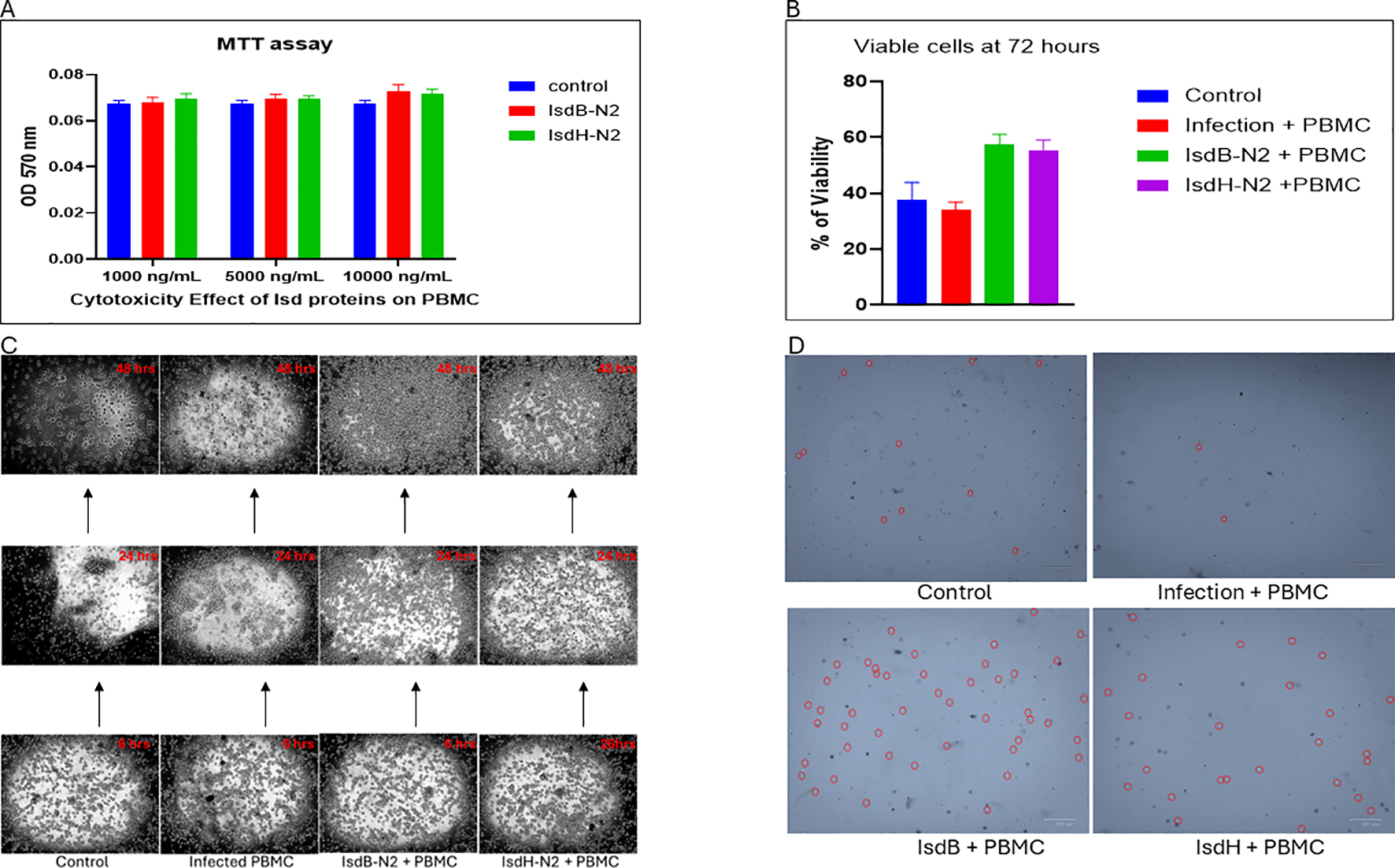
PBMC viability and proliferation following IsdB-NEAT2 and IsdH-NEAT2 exposure. **(A)** PBMCs treated with 1,000–10,000 ng/mL of either peptide showed no cytotoxicity, with viability comparable to untreated controls. **(B, C)** Both peptides induced activation and sustained PBMC responses. **(D)** Trypan blue exclusion confirmed maintained viability up to 72 h post-treatment, consistent with continued proliferation.

When PBMCs were exposed to peptide concentrations ranging from 1,000 to 10,000 ng/mL, no cytotoxic effects were observed as both IsdB-NEAT2 and IsdH-NEAT2 demonstrated an excellent safety profile *in vitro*. The PBMCs maintained normal viability across all doses tested, indicating that the peptides are well-tolerated and do not exert detrimental effects on primary immune cells within this concentration range (**Figure 5A**). Moreover, IsdB-N2 and IsdH-N2 activate and sustained PBMCs culture (**Figures 5B, C**), and remained viable for up to 72 hours, as confirmed by trypan blue (**Figure 5D**). Collectively, these results demonstrate cellular proliferation and high biocompatibility of IsdB-NEAT2 and IsdH-NEAT2, thereby supporting their suitability for downstream immunogenicity and functional analyses.

### Immunogenicity of IsdB-NEAT2 and IsdH-NEAT2 peptides

**Figure 6:**
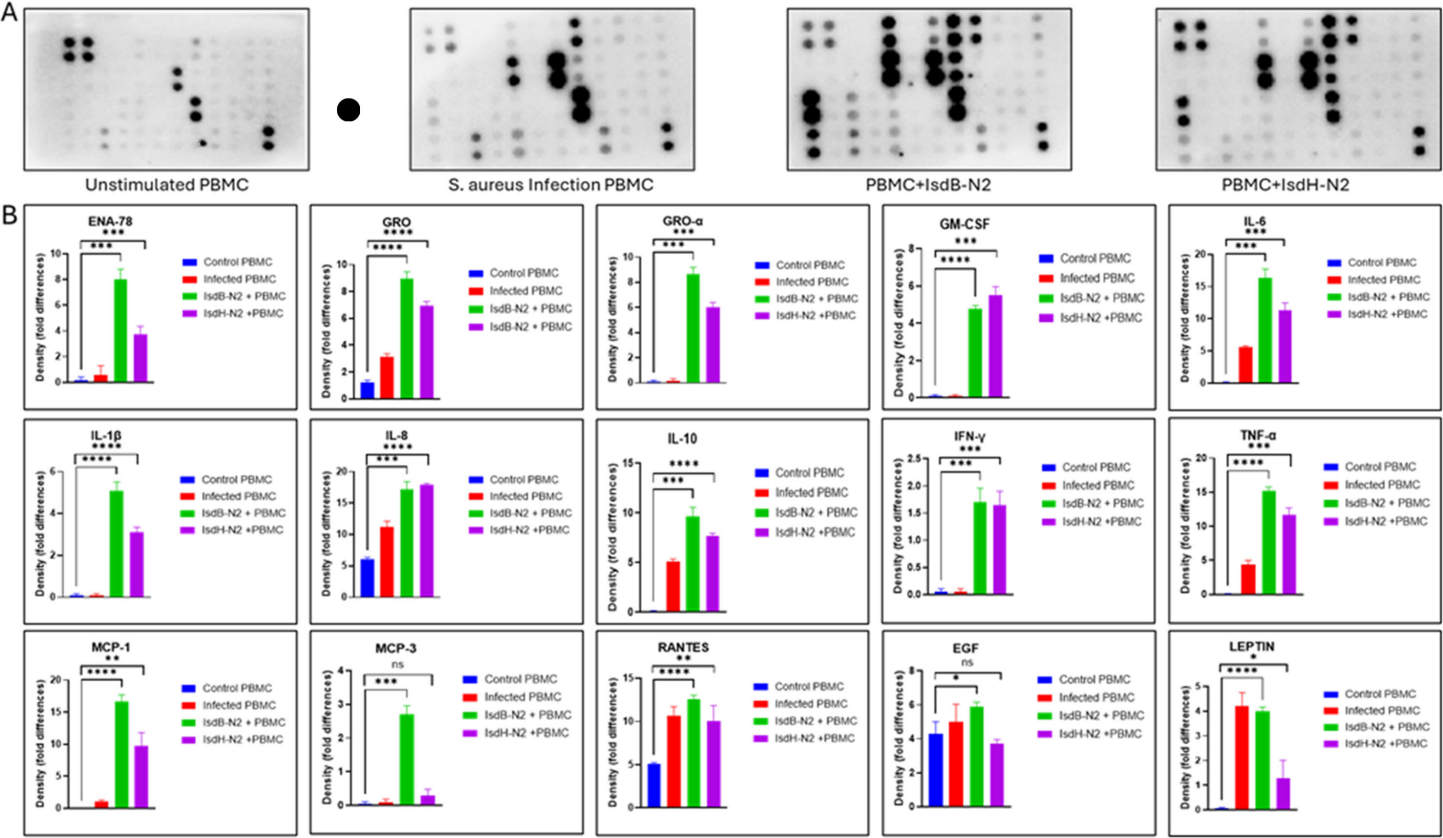
Cytokine activation following IsdB-NEAT2 and IsdH-NEAT2 stimulation. Human cytokine array analysis shows relative cytokine expression after treatment with IsdB-NEAT2, IsdH-NEAT2, or control. Both peptides increased IL-6, IL-8, IL-10, IL-1β, MCP-1, TNF-α, EGF, and RANTES compared with control, suggesting immunogenicity. IsdB-NEAT2 induced higher cytokine activation than IsdH-NEAT2, suggesting greater immunostimulatory potential. Data represents mean fold-change relative to control. *p < 0.05, **p < 0.01, ***p < 0.001 and ****p < 0.0001.

Peripheral blood mononuclear cells (PBMCs) are composed primarily of lymphocytes (T cells, B cells, and NK cells), which constitute approximately 70-90% of the population, with monocytes making up 10-20% and dendritic cells only 1-2%. To assess the immunostimulatory capacity of the synthesized peptides, we incubated PBMCs with a human cytokine array panel (**Figure 6**). Both IsdB-NEAT2 and IsdH-NEAT2 peptides induced detectable cytokine responses compared with the untreated PBMC control, demonstrating their ability to activate immune signaling pathways. Exposure to either peptide resulted in upregulation of several immune-related cytokines, including IL-6, IL-8, IL-10, IL-1β, MCP-1, TNF-α, EGF, and RANTES. Notably, IsdB-NEAT2 elicited a significantly stronger cytokine activation profile than IsdH-NEAT2, demonstrating a broader and more robust induction across both pro-inflammatory (IL-6, IL-8, TNF-α, IL-1β) and chemotactic (MCP-1, RANTES) mediators. The PBMCs from confirmed *S. aureus* MSKI stimulated immune response when compared to healthy control. The enhanced immune activation by IsdB-NEAT2 suggests that this peptide is inherently more immunogenic and capable of eliciting a stronger host immune response.

## Discussion

The present study demonstrates that the IsdB-NEAT2, IsdH-NEAT2 and SCIN peptides exhibit strong immunoreactivity with sera from patients with *Staphylococcus aureus* musculoskeletal infections (MSKI), highlighting their potential as sensitive serological biomarkers for infection detection. Elevated antigen-specific antibody responses, combined with excellent diagnostic performance reflected by high ROC-AUC values, suggest that these peptides can effectively discriminate *S. aureus* infections from both healthy individuals and MSKI caused by other etiologies. Additionally, the observed predominance of IgG responses supports the presence of an active adaptive immune response, while *in vitro* assays confirmed favorable biocompatibility and demonstrated immunogenic activity through cytokine induction, particularly for IsdB-NEAT2. Collectively, these findings preliminarily suggest that NEAT2-derived peptides may provide a promising foundation for improved serodiagnosis approaches in *S. aureus* MSKI. However, some limitations should be considered when interpreting these results, including the likely small sample size and potential variability in host immune status and infection stages.

Convectional diagnostic antigens, such as long amino acids sequences of IsdB, IsdH, SCIN and others pose several limitations due to their large molecular size, structural complexity, batch-to-batch variability, and high production costs. These limitations have hindered *S. aureus* vaccine development, compromise assay reproducibility, scalability, and affordability for diagnostic assay (4). To overcome these constraints, we designed and synthesized a short peptide containing conserved motifs unique to *S. aureus*. In this present study, the results demonstrate that our synthetic peptide retains the key antigenic determinants required for recognition of *S. aureus*, despite its significantly reduced amino acid length. This offers substantial advantages in synthesis, stability, scalability and assay specificity for the diagnosis of *S. aureus* infections.

Cytokine secretion is one of the most used outcomes for evaluating influence on immune responses and has been studied with a variety of different types of compounds (39, 40). The present study shows that the two synthesized peptides, designed from conserved functional motifs of the *S. aureus* Isd iron-acquisition system, may possess intrinsic immunogenic properties. The ability of both IsdB-NEAT2 and IsdH-NEAT2 to induce cytokine secretion suggest that these minimal antigenic domains retain their capacity to interact with immune system components, a key requirement for vaccine or diagnostic antigen development.

The markedly stronger cytokine activation observed with IsdB-NEAT2 aligns with previous reports identifying IsdB as the dominant and most immunologically relevant receptor in the Isd family (4–6). IsdB plays a central role in heme acquisition; a critical step for *S. aureus* survival in the host, making it more exposed and more frequently encountered by the immune system during infection. The robust induction of IL-6, TNF-α, and IL-1β suggests that IsdB-NEAT2 stimulates innate immune pathways associated with inflammatory activation and early pathogen recognition. Likewise, the heightened levels of chemokines such as MCP-1 and RANTES support enhanced recruitment of monocytes and T cells, reflecting an ability to initiate downstream adaptive responses.

### Implications for future applications

The stronger immunogenic profile of both peptides (IsdB and IsdH) but more importantly IsdB-NEAT2 supports its potential as a superior antigen candidate compared with IsdH-NEAT2. Given the minimal domain design, these peptides offer advantages in stability, ease of synthesis, and reduced risk of off-target responses. The finding that small, functional motifs can trigger significant immune activation underscores their applicability for targeting *S. aureus* iron-uptake mechanisms. Accordingly, this peptide represents a promising candidate for development as a diagnostic biomarker and warrants further investigation in the context of immunological characterization, adjuvant optimization, and *in vivo* protection studies.

## Conclusions

In summary, this study establishes that rationally designed short peptides derived from conserved NEAT2 domains of key *Staphylococcus aureus* virulence factors IsdB, and IsdH retain antigenicity and immunogenicity despite their minimal structural composition. These peptides offer significant advantages in synthesis simplicity, cost-effectiveness, specificity, and adaptability to multiple diagnostic formats. Its conserved nature across *S. aureus* strains reinforces its reliability as molecular markers. These preliminary findings, while derived from data that remain subject to further validation and comprehensive analysis, provide suggestive evidence that key antigenic determinants necessary for immune recognition may be conserved within structurally compact functional motifs. Furthermore, the observed cytokine induction profile particularly the activation of pro-inflammatory mediators alongside chemokines involved in immune cell recruitment highlights the biological relevance of these peptides and supports their potential utility in diagnostics and immunomodulatory platforms. Nevertheless, these findings should be interpreted within the context of key limitations.

### Limitations

This study should be interpreted considering the relatively small cohort sample size, which represents a key methodological limitation. It should be noted that our findings of Isd peptides performance are a preliminary model based on semi quantitative that warrant further investigations. Although an elevated AUC suggests strong discriminatory performance of the peptides, limited sample size increases the risk of overfitting and may inflate performance estimates due to reduced variability and potential sampling bias. Therefore, while the high AUC, IgM/IgG, PBMC and cytokines activation supports the potential serodiagnosis utility of these short peptides, these findings should be considered preliminary. External validation in larger, well-characterized populations is necessary to confirm the stability of the AUC and to refine clinically applicable cut-off thresholds.

## Data Availability

The raw data supporting the conclusions of this article will be made available by the authors, without undue reservation.

## Supplementary material

Supplementary Figure 1 Predicted antigenic sites on IsdB-N2. Primary sequence (N→C) and AlphaFold3-predicted monomeric structure of amino acids and BepiPred2.0 mapped antigenic regions. Residues are highlighted on the surface-rendered structure in distinct colors, with 90° and 180° rotated views provided.

Supplementary Figure 2 Predicted antigenic sites on IsdH-N2. Primary sequence (N→C) and AlphaFold3-predicted monomeric structure of amino acids and BepiPred2.0 mapped antigenic regions. Residues are highlighted on the surface-rendered structure in distinct colors, with 90° and 180° rotated views provided.

## References

[1] Linz MS Mattappallil A Finkel D Parker D . Clinical impact of Staphylococcus aureus skin and soft tissue infections. Antibiot (Basel). (2023) 12(3):557. doi: 10.3390/antibiotics12030557. PMID: 36978425 PMC10044708

[2] Lee BY Singh A David MZ Bartsch SM Slayton RB Huang SS . The economic burden of community-associated methicillin-resistant Staphylococcus aureus (CA-MRSA). Clin Microbiol Infect. (2013) 19:528–36. doi: 10.1111/j.1469-0691.2012.03914.x. PMID: 22712729 PMC3463640

[3] Touaitia R Mairi A Ibrahim NA Basher NS Idres T Touati A . Staphylococcus aureus: a review of the pathogenesis and virulence mechanisms. Antibiot (Basel). (2025) 14(5):470. doi: 10.3390/antibiotics14050470. PMID: 40426537 PMC12108373

[4] Chiang AWT Tsai C-H Li H Díez ML Gonzalez C Trieu D . Non-protective immune imprint underlies failure of Staphylococcus aureus IsdB vaccine. Cell Host Microbe. (2022) 30:1163–72:e1166. doi: 10.1016/j.chom.2022.06.006. PMID: 35803276 PMC9378590

[5] Muthukrishnan G Masters EA Daiss JL Schwarz EM . Mechanisms of immune evasion and bone tissue colonization that make Staphylococcus aureus the primary pathogen in osteomyelitis. Curr Osteoporos Rep. (2019) 17:395–404. doi: 10.1007/s11914-019-00548-4. PMID: 31721069 PMC7344867

[6] Oh I Muthukrishnan G Ninomiya MJ Brodell JD Smith BL Lee CC . Tracking anti-Staphylococcus aureus antibodies produced *In Vivo* and Ex Vivo during foot salvage therapy for diabetic foot infections reveals prognostic insights and evidence of diversified humoral immunity. Infect Immun. (2018) 86. doi: 10.1128/iai.00629-18 PMC6246899 30275008

[7] Becker K Heilmann C Peters G . Coagulase-negative staphylococci. Clin Microbiol Rev. (2014) 27:870–926. doi: 10.1128/cmr.00109-13. PMID: 25278577 PMC4187637

[8] Mairi A Ibrahim NA Idres T Touati A . A comprehensive review of detection methods for Staphylococcus aureus and its enterotoxins in food: from traditional to emerging technologies. Toxins (Basel). (2025) 17(7):319. doi: 10.3390/toxins17070319. PMID: 40711131 PMC12298662

[9] Li Q Dou L Zhang Y Luo L Yang H Wen K . A comprehensive review on the detection of Staphylococcus aureus enterotoxins in food samples. Compr Rev Food Sci Food Saf. (2024) 23:e13264. doi: 10.1111/1541-4337.13264. PMID: 38284582

[10] Duan R Wang P . Rapid and simple approaches for diagnosis of Staphylococcus aureus in bloodstream infections. Pol J Microbiol. (2022) 71:481–9. doi: 10.33073/pjm-2022-050. PMID: 36476633 PMC9944965

[11] MaChado I Goikoetxea G Alday E Jiménez T Arias-Moreno X Hernandez FJ . Ultra-sensitive and specific detection of S. aureus bacterial cultures using an oligonucleotide probe integrated in a lateral flow-based device. Diagn (Basel). (2021) 11. doi: 10.3390/diagnostics11112022. PMID: 34829369 PMC8619029

[12] Srisrattakarn A Charoensri N Prompipak J Ouancharee W Saiboonjan B Tippayawat P . Rapid detection of Staphylococcus aureus in blood culture samples using human IgG-based lateral flow assay. Microbiol Spectr. (2024) 12:e0304623. doi: 10.1128/spectrum.03046-23. PMID: 38230955 PMC10846088

[13] Tarisse CF Goulard-Huet C Nia Y Devilliers K Marcé D Dambrune C . Highly sensitive and specific detection of staphylococcal enterotoxins SEA, SEG, SEH, and SEI by immunoassay. Toxins (Basel). (2021) 13(2):130. doi: 10.3390/toxins13020130. PMID: 33572449 PMC7916246

[14] Nairz M Dichtl S Schroll A Haschka D Tymoszuk P Theurl I . Iron and innate antimicrobial immunity-depriving the pathogen, defending the host. J Trace Elem Med Biol. (2018) 48:118–33. doi: 10.1016/j.jtemb.2018.03.007. PMID: 29773170

[15] Hammer ND Skaar EP . Molecular mechanisms of Staphylococcus aureus iron acquisition. Annu Rev Microbiol. (2011) 65:129–47. doi: 10.1146/annurev-micro-090110-102851 PMC3807827 21639791

[16] Cheung J Beasley FC Liu S Lajoie GA Heinrichs DE . Molecular characterization of staphyloferrin B biosynthesis in Staphylococcus aureus. Mol Microbiol. (2009) 74:594–608. doi: 10.1111/j.1365-2958.2009.06880.x. PMID: 19775248

[17] Laakso HA Marolda CL Pinter TB Stillman MJ Heinrichs DE . A heme-responsive regulator controls synthesis of staphyloferrin B in Staphylococcus aureus. J Biol Chem. (2016) 291:29–40. doi: 10.1074/jbc.m115.696625. PMID: 26534960 PMC4697164

[18] van Dijk MC de Kruijff RM Hagedoorn PL . The role of iron in Staphylococcus aureus infection and human disease: a metal tug of war at the host-microbe interface. Front Cell Dev Biol. (2022) 10:857237. doi: 10.3389/fcell.2022.857237. PMID: 35399529 PMC8986978

[19] Valenciano-Bellido S Caaveiro JMM Nakakido M Nakakido M Kuroda D Aikawa C . Targeting hemoglobin receptors IsdH and IsdB of Staphylococcus aureus with a single VHH antibody inhibits bacterial growth. J Biol Chem. (2023) 299:104927. doi: 10.1016/j.jbc.2023.104927. PMID: 37330175 PMC10466926

[20] Conroy BS Grigg JC Kolesnikov M Morales LD Michael Murphy ME . Staphylococcus aureus heme and siderophore-iron acquisition pathways. Biometals. (2019) 32:409–24. doi: 10.1007/s10534-019-00188-2. PMID: 30911924

[21] Skaar EP Schneewind O . Iron-regulated surface determinants (Isd) of Staphylococcus aureus: stealing iron from heme. Microbes Infect. (2004) 6:390–7. doi: 10.1016/j.micinf.2003.12.008. PMID: 15101396

[22] Maresso AW Schneewind O . Iron acquisition and transport in Staphylococcus aureus. Biometals. (2006) 19:193–202. doi: 10.1007/s10534-005-4863-7. PMID: 16718604

[23] Skaar EP . The battle for iron between bacterial pathogens and their vertebrate hosts. PLoS Pathog. (2010) 6:e1000949. doi: 10.1371/journal.ppat.1000949. PMID: 20711357 PMC2920840

[24] Grigg JC Ukpabi G Gaudin CF Murphy ME . Structural biology of heme binding in the Staphylococcus aureus Isd system. J Inorg Biochem. (2010) 104:341–8. doi: 10.1016/j.jinorgbio.2009.09.012. PMID: 19853304

[25] Pilpa RM Fadeev EA Villareal VA Wong ML Phillips M Clubb RT . Solution structure of the NEAT (NEAr Transporter) domain from IsdH/HarA: the human hemoglobin receptor in Staphylococcus aureus. J Mol Biol. (2006) 360:435–47. doi: 10.1016/j.jmb.2006.05.019. PMID: 16762363

[26] Pishchany G Sheldon JR Dickson CF Alam MT Read TD Gell DA . IsdB-dependent hemoglobin binding is required for acquisition of heme by Staphylococcus aureus. J Infect Dis. (2014) 209:1764–72. doi: 10.1093/infdis/jit817. PMID: 24338348 PMC4038968

[27] Ellis-Guardiola K Clayton J Pham C Mahoney BJ Wereszczynski J Clubb RT . The Staphylococcus aureus IsdH receptor forms a dynamic complex with human hemoglobin that triggers heme release via two distinct hot spots. J Mol Biol. (2020) 432:1064–82. doi: 10.1016/j.jmb.2019.12.023. PMID: 31881209 PMC7309296

[28] Yang S Wang M Wang T Sun M Huang H Shi X . Self-assembled short peptides: recent advances and strategies for potential pharmaceutical applications. Mater Today Bio. (2023) 20:100644. doi: 10.1016/j.mtbio.2023.100644. PMID: 37214549 PMC10199221

[29] Adler-Abramovich L Gazit E . The physical properties of supramolecular peptide assemblies: from building block association to technological applications. Chem Soc Rev. (2014) 43:6881–93. doi: 10.1039/c4cs00164h. PMID: 25099656

[30] Pandey S Malviya G Chottova Dvorakova M . Role of peptides in diagnostics. Int J Mol Sci. (2021) 22(16):8828. doi: 10.3390/ijms22168828. PMID: 34445532 PMC8396325

[31] Gomara MJ Haro I . Synthetic peptides for the immunodiagnosis of human diseases. Curr Med Chem. (2007) 14:531–46. doi: 10.2174/092986707780059698. PMID: 17346145

[32] Vengesai A Kasambala M Mutandadzi H Mduluza-Jokonya TL Mduluza T Naicker T . Scoping review of the applications of peptide microarrays on the fight against human infections. PLoS One. (2022) 17:e0248666. doi: 10.1371/journal.pone.0248666. PMID: 35077448 PMC8789108

[33] Pietrocola G Pellegrini A Alfeo MJ Marchese L Foster TJ Speziale P . The iron-regulated surface determinant B (IsdB) protein from Staphylococcus aureus acts as a receptor for the host prote*in vitro*nectin. J Biol Chem. (2020) 295:10008–22. doi: 10.1074/jbc.ra120.013510. PMID: 32499371 PMC7380192

[34] Cretich M Gori A D'Annessa I Chiari M Colombo G . Peptides for infectious diseases: from probe design to diagnostic microarrays. Antibodies (Basel). (2019) 8(1):23. doi: 10.3390/antib8010023. PMID: 31544829 PMC6640701

[35] Pilpa RM Robson SA Villareal VA Wong ML Phillips M Clubb RT . Functionally distinct NEAT (NEAr Transporter) domains within the Staphylococcus aureus IsdH/HarA protein extract heme from methemoglobin. J Biol Chem. (2009) 284:1166–76. doi: 10.1074/jbc.m806007200. PMID: 18984582 PMC2613621

[36] Nishitani K Ishikawa M Morita Y Yokogawa N Xie C Bentley KL . IsdB antibody-mediated sepsis following S. aureus surgical site infection. JCI Insight. (2020) 5(19):e141164. doi: 10.1172/jci.insight.141164. PMID: 33004694 PMC7566716

[37] Bennett MR Dong J Bombardi RG Soto C Parrington HM Nargi RS . Human V(H)1–69 gene-encoded human monoclonal antibodies against Staphylococcus aureus IsdB use at least three distinct modes of binding to inhibit bacterial growth and pathogenesis. mBio. (2019) 10(5):e02473–19. doi: 10.1201/9780203912065.ch4 PMC6805997 31641091

[38] Spirig T Malmirchegini GR Zhang J Robson SA Sjodt M Liu M . Staphylococcus aureus uses a novel multidomain receptor to break apart human hemoglobin and steal its heme. J Biol Chem. (2013) 288:1065–78. doi: 10.1074/jbc.m112.419119. PMID: 23132864 PMC3542992

[39] Schroecksnadel S Gostner J Jenny M Kurz K Schennach H Weiss G . Immunomodulatory effects *in vitro* of vitamin K antagonist acenocoumarol. Thromb Res. (2013) 131:e264-269. doi: 10.1016/s0049-3848(12)70091-9. PMID: 23481478

[40] Kouakou K Schepetkin IA Yapi A Kirpotina LN Jutila MA Quinn MT . Immunomodulatory activity of polysaccharides isolated from Alchornea cordifolia. J Ethnopharmacol. (2013) 146:232–42. doi: 10.1016/j.jep.2012.12.037. PMID: 23291534 PMC3577965

