# Supplementary figures and images for: Synthetic short peptide mimicking IsdB and IsdH conserved motifs selectively bind and enable detection of Staphylococcus aureus infection
Source: Front Immunol. 2026 May 20;17:1813440. doi: 10.3389/fimmu.2026.1813440 (PMC13229835; doi:10.3389/fimmu.2026.1813440)

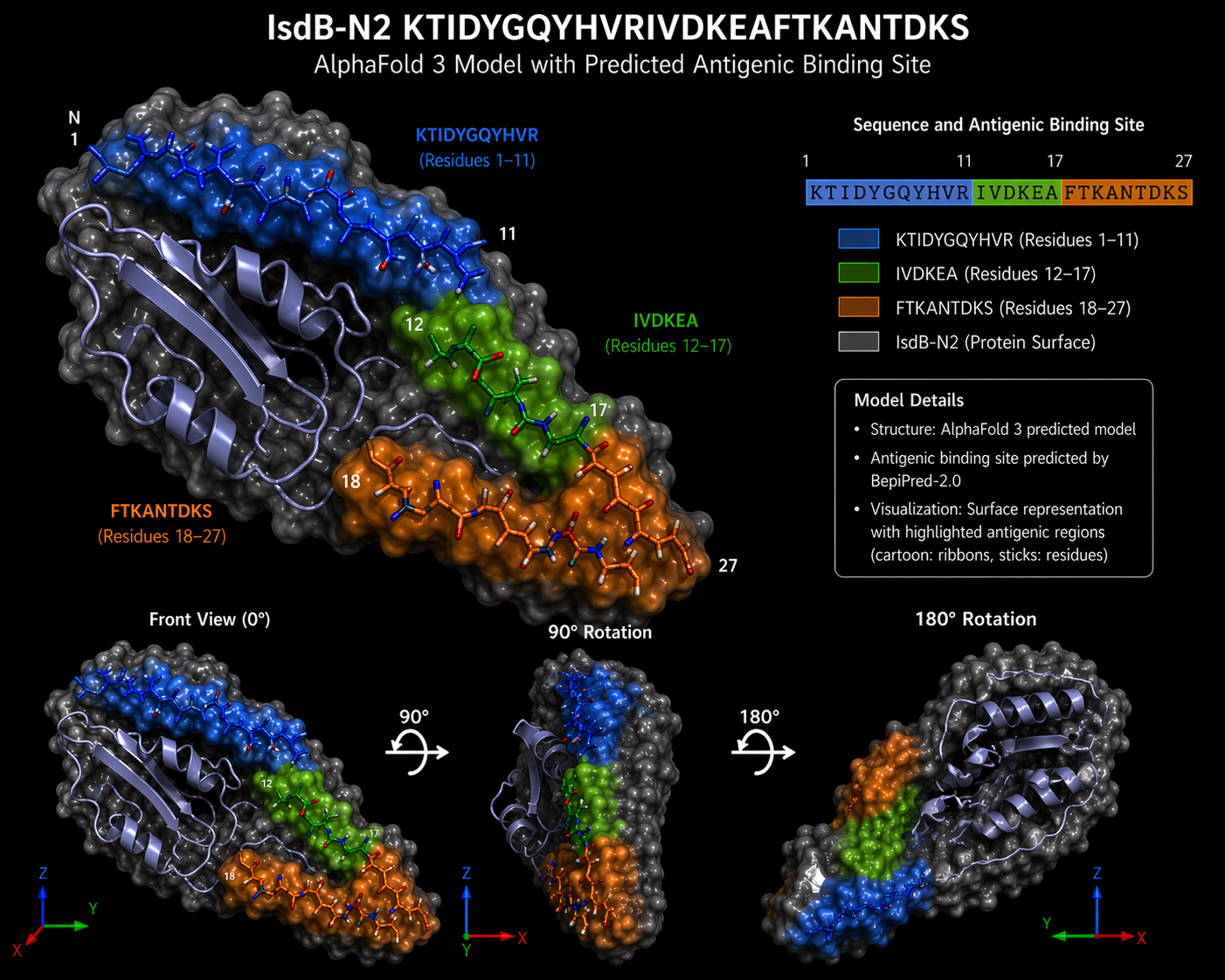

Supplement: Supplementary Figure 1 — Predicted antigenic sites on IsdB-N2. Primary sequence (N→C) and AlphaFold3-predicted monomeric structure of amino acids and BepiPred2.0 mapped antigenic regions. Residues are highlighted on the surface-rendered structure in distinct colors, with 90° and 180° rotated views provided. [file Image1.tiff]

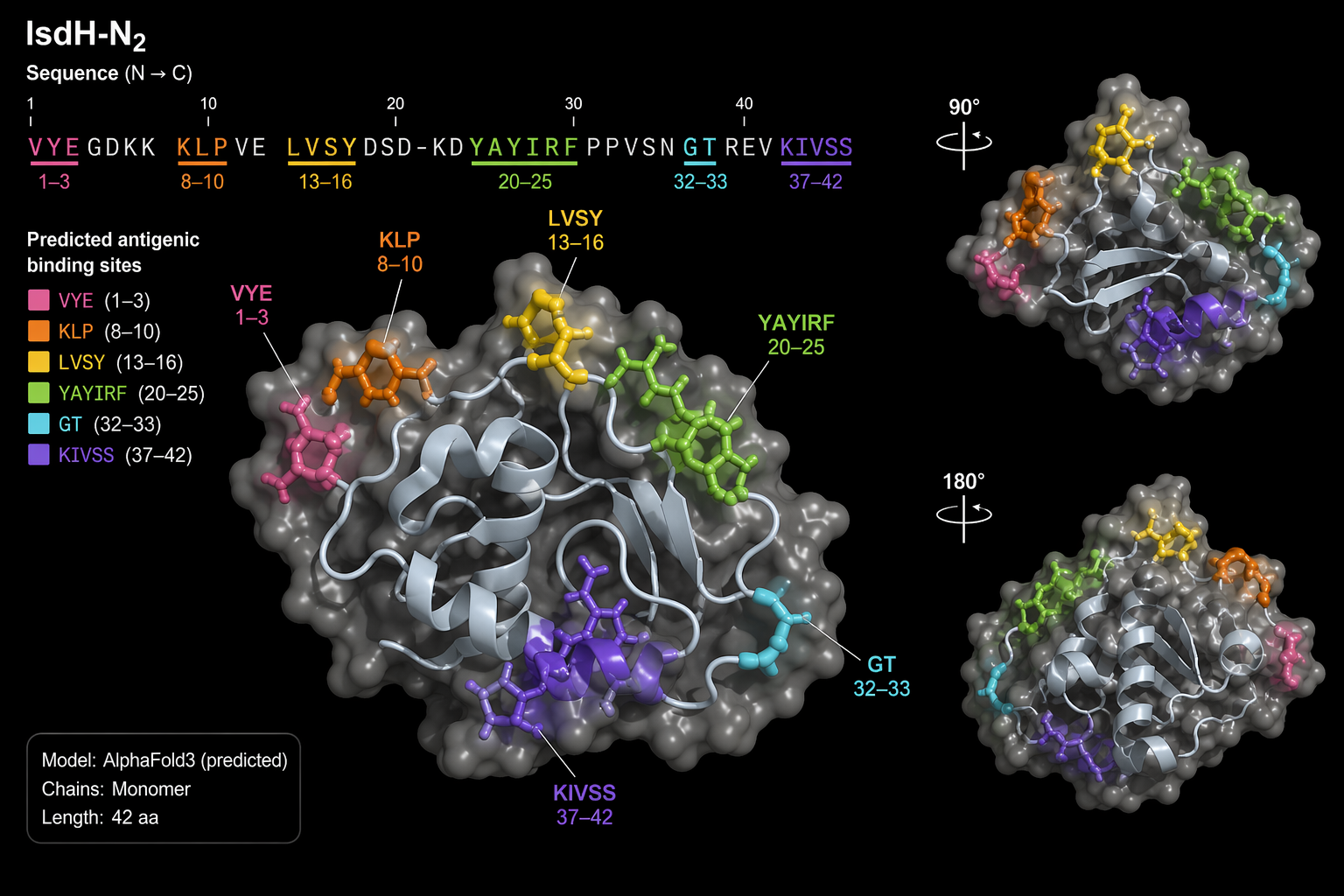

Supplement: Supplementary Figure 2 — Predicted antigenic sites on IsdH-N2. Primary sequence (N→C) and AlphaFold3-predicted monomeric structure of amino acids and BepiPred2.0 mapped antigenic regions. Residues are highlighted on the surface-rendered structure in distinct colors, with 90° and 180° rotated views provided. [file Image2.tiff]
